# Supplementary material for: Influenza A Virus Infection Reactivates Human Endogenous Retroviruses Associated with Modulation of Antiviral Immunity
Source: Viruses. 2022 Jul 21;14(7):1591. doi: 10.3390/v14071591 (PMC9320126; doi:10.3390/v14071591)
Supplement: Supplementary file 1 [file viruses-14-01591-s001.zip › MotifAnalysis.zip!/MotifOutput_SC35MdNS1/homerResults/motif9.similar.html]

motif9

## Information for motif9

C
T
A
G
A
C
G
T
A
C
T
G
C
T
A
G
C
T
A
G
A
C
T
G
G
C
T
A
A
G
T
C
A
C
G
T
A
G
C
T
  
Reverse Opposite:  

C
T
G
A
G
T
C
A
A
C
T
G
C
G
A
T
A
G
T
C
A
G
T
C
A
G
T
C
A
G
T
C
C
G
T
A
A
G
T
C
  

|  |  |
| --- | --- |
| p-value: | 1e-35 |
| log p-value: | -8.270e+01 |
| Information Content per bp: | 1.882 |
| Number of Target Sequences with motif | 26.0 |
| Percentage of Target Sequences with motif | 33.77% |
| Number of Background Sequences with motif | 313.4 |
| Percentage of Background Sequences with motif | 0.69% |
| Average Position of motif in Targets | 84.4 +/- 51.6bp |
| Average Position of motif in Background | 97.5 +/- 82.9bp |
| Strand Bias (log2 ratio + to - strand density) | 3.8 |
| Multiplicity (# of sites on avg that occur together) | 1.07 |
| Motif File: | file (matrix) reverse opposite |

### Similar de novo motifs found

|  |  |  |  |  |  |  |  |
| --- | --- | --- | --- | --- | --- | --- | --- |
| Rank | Match Score | Redundant Motif | P-value | log P-value | % of Targets | % of Background | Motif file |
| 1 | 0.797 | A C T G A T C G A C T G A C T G G T C A G T A C C A T G A C G T | 1e-26 | -60.499527 | 32.47% | 1.44% | motif file (matrix) |
| 2 | 0.708 | A C T G A C G T A C T G C G T A A C T G A C T G C G T A A G T C | 1e-13 | -30.363674 | 24.68% | 2.55% | motif file (matrix) |
| 3 | 0.613 | A T C G A T G C C T A G G T A C C A T G C T A G A T G C G T A C G A C T A C G T | 1e-12 | -27.928737 | 12.99% | 0.39% | motif file (matrix) |
